# Small molecule-mediated targeting of microRNAs for drug discovery: Experiments, computational techniques, and disease implications

**DOI:** 10.1016/j.ejmech.2023.115500

**Published:** 2023-09-05

**Authors:** Jianfeng Sun, Miaoer Xu, Jinlong Ru, Anna James-Bott, Dapeng Xiong, Xia Wang, Adam P. Cribbs

**Affiliations:** a Botnar Research Centre, Nuffield Department of Orthopedics, Rheumatology and Musculoskeletal Sciences, University of Oxford, Oxford, OX3 7LD, UK; b Department of Biology, Emory University, Atlanta, GA, 30322, USA; c Chair of Prevention of Microbial Diseases, School of Life Sciences Weihenstephan, Technical University of Munich, Freising, 85354, Germany; d Department of Computational Biology, Cornell University, Ithaca, NY, 14853, USA; e Weill Institute for Cell and Molecular Biology, Cornell University, Ithaca, NY, 14853, USA; f College of Animal Science and Technology, Northwest A&F University, Yangling, 712100, China

**Keywords:** microRNA targets, Drug discovery, Molecular interactions, Disease implications, Deep learning

## Abstract

Small molecules have been providing medical breakthroughs for human diseases for more than a century. Recently, identifying small molecule inhibitors that target microRNAs (miRNAs) has gained importance, despite the challenges posed by labour-intensive screening experiments and the significant efforts required for medicinal chemistry optimization. Numerous experimentally-verified cases have demonstrated the potential of miRNA-targeted small molecule inhibitors for disease treatment. This new approach is grounded in their posttranscriptional regulation of the expression of disease-associated genes. Reversing dysregulated gene expression using this mechanism may help control dysfunctional pathways. Furthermore, the ongoing improvement of algorithms has allowed for the integration of computational strategies built on top of laboratory-based data, facilitating a more precise and rational design and discovery of lead compounds. To complement the use of extensive pharmacogenomics data in prioritising potential drugs, our previous work introduced a computational approach based on only molecular sequences. Moreover, various computational tools for predicting molecular interactions in biological networks using similarity-based inference techniques have been accumulated in established studies. However, there are a limited number of comprehensive reviews covering both computational and experimental drug discovery processes. In this review, we outline a cohesive overview of both biological and computational applications in miRNA-targeted drug discovery, along with their disease implications and clinical significance. Finally, utilizing drug-target interaction (DTIs) data from DrugBank, we showcase the effectiveness of deep learning for obtaining the physicochemical characterization of DTIs.

## Highlights

• We provide a cohesive overview of both biological and computational applications in discovering drugs of miRNA targets.
• We conduct a benchmark on mechanisms and experiments of small molecule-mediated targeting of miRNAs for cancer therapeutics.
• We review a deep learning-assisted drug repositioning scheme made based on connectivity scores and DeepsmirUD predictions.
• We summarize the current status of the computational discovery of drugs of miRNA and protein targets.
• We present a few convolution-based deep learning architectures for predicting regulation types of small molecules and miRNAs.

## Graphical abstract

**Figure:**
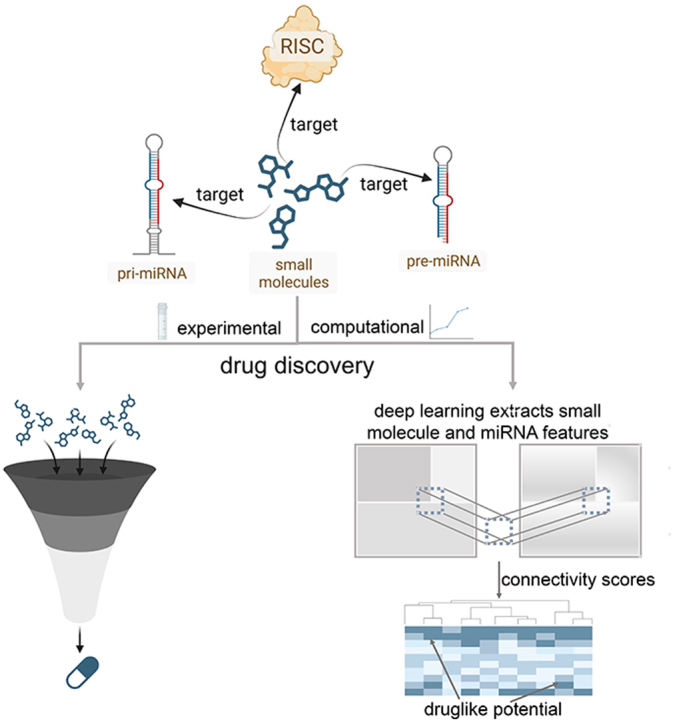

## 1 Introduction

Small molecules have long been recognized as a cornerstone in drug development for treating a wide variety of human diseases [1,2]. As of January 2023, the DrugBank database (current version: 5.1.10) has compiled 2736 small molecule drugs that are approved by the Food and Drug Administration (FDA), with over 4500 in preclinical or animal testing stages [3]. Recently, there has been a surge in interest in discovering small molecule inhibitors that specifically target noncoding RNAs (ncRNAs) [4,5].

Protein-coding genes make up about 1.5% of the human genome [6,7], of which only 3–4% is considered druggable targets [8]. The vast majority of undruggable proteins pose challenges for disease treatment, prompting researchers to explore targeting ncRNAs, especially, to address the issues on dysregulated post-transcriptional regulation [9,10]. ncRNAs are a class of RNA molecules that do not encode proteins, constituting around 70% of the eukaryote transcriptome [8]. Taking into account regions like introns and 3′ and 5′ untranslated regions, the proportion of non-coding RNA molecules can encompass approximately 95–98.5% of the total RNA proportion in a cell [7,11,12]. The extensive presence of these RNA molecules, combined with their important roles in gene regulation, makes them attractive drug targets [13]. A major class of ncRNAs is microRNAs (miRNAs), which are crucial for regulating cellular activities [14]. Small molecule-mediated targeting of miRNAs can occur before and after miRNA maturation. More recently, research by Childs-Disney et al. has advanced our understanding of the small molecule-mediated targeting of miRNAs by identifying inhibitors that bind to the tertiarily folded structures of miRNAs [15]. Subsequently, several other molecules have been identified that work using a similar mechanism [8,[16], [17], [18]], leading to a deeper understanding of small-molecule RNA binding. This understanding has been particularly useful in elucidating how stem-loop hairpin secondary structures are folded into tertiary structures, resulting in the formation of pockets or bulges (such as those found near the Dicer processing site [17]) that aid in small molecule interactions. As a result of this enhanced understanding of small molecule-mediated targeting of miRNAs at the molecular level, researchers have been able to pursue computational rational design for lead compounds and develop algorithms to predict small molecule-miRNA interactions.

Computational approaches for small molecule drug discovery can be broadly categorized into two groups from a structural perspective, based on whether they leverage information about the structure-activity relationships of small molecules [19] and/or the structural details of miRNA targets. In addition to establishing connections between diseases and small molecules, approaches that incorporate such information can also partly or fully facilitate lead compound design [20]. For instance, Inforna has pioneered a novel strategy for discovering small molecule drugs targeting oncogenic miRNA targets by mining from a large dataset of RNA-motif small-molecule interaction pairs, sourced from a microarray-screened library [21]. Specifically, it uses predicted structure annotations of a miRNA of interest to probe highly similar RNA motifs that interact with small molecules. Then, through an algorithmic scoring scheme, the potential small molecules are prioritized for producing lead compounds that are mostly like to treat cancer [22]. On the other hand, the second category often utilizes the information about molecular sequences or simply drug-target as well as target-disease relationships for drug screening [23,24], which can be achieved by using a variety of similarity-based inference methods [25] or machine learning methods [26]. Recently, we developed deep learning approaches to predict the specific regulation types between a small molecule and a miRNA, relying solely on molecular sequence information [27]. Subsequently, we implemented a drug repositioning strategy that connects a drug and a disease using calculated connectivity scores through the overlapped disease and drug relationships with miRNAs (as in Ref. [28]). These connectivity scores were introduced to facilitate drug repositioning based on distinct upregulation and downregulation information [29], which was obtained through statistical tests using gene/transcriptional expression data from large-scale pharmacogenomic studies [30,31]. Nonetheless, the scarcity of miRNA expression data associated with numerous small molecules makes it difficult to address using existing techniques, as generating such data requires considerable experimental effort. In this context, computational strategies offer an alternative solution to readily generate information about miRNA regulation.

In this review, we attempt to summarize the small molecule-mediated targeting strategies for the discovery of drugs for both protein and miRNA targets. We cover the functional importance of miRNAs as small-molecule targets, disease implications, clinical applications, and miRNA pharmacogenomics. We also benchmark three key prediction problems in early drug discovery: predicting small molecule-miRNA (SM-miR) associations [32], SM-miR regulation types [27], and drug-target interactions (DTIs) [33]. To assess the current status of the DTI prediction based on sequences alone, we analysed the DrugBank database using deep learning methods, achieving an area under the curve (AUC) of approximately 95% for independent test DTIs and 75% for those involving novel drugs. Recognizing the transformative potential of artificial intelligence, we additionally provide an overview of key deep learning methodologies in DTI prediction [34,35]. Our review complements existing literature by integrating experimental and computational aspects of drug discovery.

## 2 miRNA dysregulation in diseases

Due to their critical role in gene expression regulation, miRNAs have been implicated in a wide spectrum of diseases [14,36]. For example, the overexpression of miR-17–92, a widely studied miRNA cluster [37], is found to result in inhibiting proximal epithelial cell differentiation [38] and polycystic kidney disease (PKD) [39]. Regulus Therapeutics has developed a first-in-class oligonucleotide-based drug RGLS4326 to treat PKD by targeting miR-17, a member of the miR-17–92 family [37]. Its clinical trials are currently underway [40,41]. Also, long noncoding RNAs (lncRNAs), another major component of ncRNAs (>100,000 human lncRNAs [42]), are highly associated with a large number of human diseases [43,44]. For example, the LncRNADisease database (v2.0) has currently compiled 10,002 lncRNA-disease associations that are detected in humans, which account for 94.7% of all the associations [45]. Dysregulated expression levels of multiple miRNAs are often observed in a single disease. For example, Yanaihara et al. conducted a correlation analysis of miRNA expression profiles in patients with lung adenocarcinoma and discovered the aberrant expression of several miRNAs, such as the upregulation of mir-155, the downregulation of let-7a-2, and the downregulation of mir-145. The expression of these miRNAs was found to be correlated with the patient survival time [46]. miRNAs can also work in conjunction with other types of molecules to exert and amplify oncogenic effects in diseases. For example, the Myc oncogene (encoding a transcription factor in charge of transcription events of ∼15% genes [47]) and miR-155 have been seen to be co-overexpressed to promote B-cell lymphomas [48]. These findings imply that the complex interplay of a combination of dysregulated miRNAs may contribute to the severity of certain diseases.

## 3 Small molecules targeting miRNAs for cancer therapeutics

**Fig. 1:**
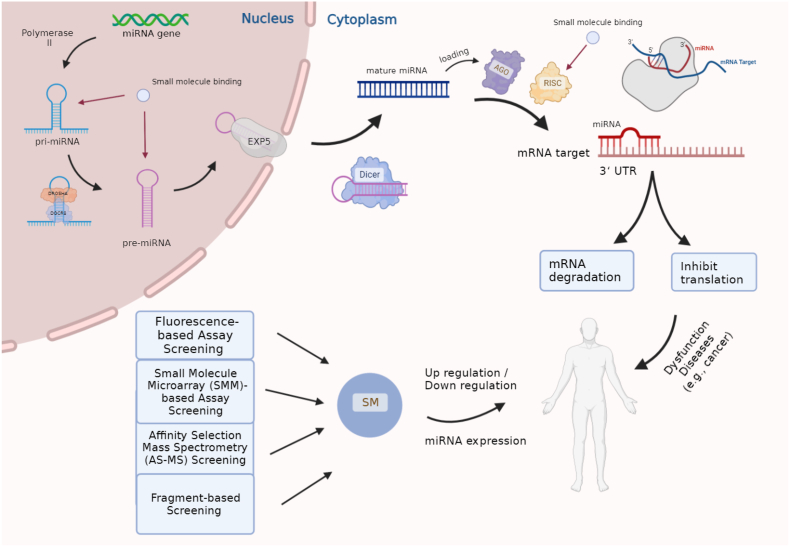
Small molecule therapeutics targeting the biogenesis of miRNAs. In the cell nucleus, miRNA genes are first transcribed by RNA polymerase II into primary transcripts (pri-miRNAs) whose monocistronic, polycistronic, and/or intronic regions (see a comprehensive review [249]) possess the characteristic hairpin secondary structures [51] in which mature miRNA sequences are located [250]. After being recognized by DGCR8 (i.e., DiGeorge critical region 8, the RNA-binding protein) [251] and Drosha (i.e., the RNase III enzyme) [252], these hairpin structures are then cleaved to produce the precursor miRNAs, known as pre-miRNAs [253]. Subsequently, the pre-miRNAs are transported by exportin-5 [254] to the cytoplasm, in which they are converted into mature miRNAs through the Dicer-mediated processing mechanism [245,255]. The mature miRNAs culminate in being loaded with the Argonaute (AGO) protein into a bioactive miRNA-induced silencing complex (miRISC). The loaded miRNAs will guide miRISCs to initiate its binding to the complementary sequences in the 3′untranslated region (UTR) of their mRNA targets [256]. The result of miRNA binding is the silencing of the target gene [58], which occurs in a phased manner over time [256]. During the early phase, the target gene's translation is inhibited [249], while in the later phase, mRNAs transcribed by the affected genes are degraded through mechanisms such as mRNA deadenylation [257]. This phased progress gives miRNAs two distinct roles: the inhibition of gene translation and the degradation of mRNAs [258]. The dysregulation of the expression of the miRNAs will cause the onset of their regulated disease pathways, while the small molecule identified as being helpful for inhibition can be used to rectify the dysregulation of the miRNA expression for disease treatment. For example, this can be achieved by targeting pri-miRNAs and pre-miRNAs within the nucleus and miRISCs in the cytoplasm.

Physical interactions of multiple kinds of biological molecules with miRNAs of interest provide the potential to alter the functions of the miRNAs, which has made it possible to regulate their controlled genes that are abnormally expressed in human diseases. This has opened up extensive possibilities for developing miRNA targeting strategies using small molecules [4], miRNA sponges [49], or antisense oligonucleotides [50]. The efficacy of using oligonucleotides and small molecules is discussed in detail as in Refs. [17,21], which indicate that small molecules possess several advantages over oligonucleotides, such as greater selectivity for inhibition and increased cell permeability under specific conditions. Our review is centred on the targeting of miRNAs by small molecules. Rather than directly inhibiting disease-related gene targets, small molecules achieve therapeutic effects by regulating the transcriptional expression of miRNAs, which subsequently affects the expression of malfunctioning gene targets indirectly [4]. Small molecule-mediated regulation of miRNAs can occur at many stages over the course of miRNA biogenesis (see Fig. 1). Specifically, small molecules can achieve therapeutic effects not only by directly binding to mature miRNAs but also by binding to the functional sites (such as DROSHA processing sites) in miRNA precursors where mature miRNAs are located, inhibiting the biogenesis of miRNAs, as suggested by Childs-Disney et al. [15]. Aside from targeting the functional sites, Li and Rana described two additional methods for regulating miRNA expression: blockading the assembly of pri-miRNAs and obstructing the formation of the miRNA-induced silencing complex (miRISC) [4], which affect the miRNA maturation and mRNA degradation processes, respectively [51]. Similar to disrupting the miRISC, small molecules could target other miRNA-binding proteins (miRBPs) to modulate miRNA expression, such as LIN28 [52], Toll-like receptors [53], and non-canonical bacterial RBPs [16]. The mechanism of small molecule therapeutics is self-explanatory, as they can induce a change in gene expression profiles in diseases by targeting miRNAs, irrespective of whether the altered miRNA expression arises from mRNA degradation or miRNA assembly processes.

## 4 Experimental determination of interactions between small molecules and miRNAs

The increasing number of experimentally-evidenced cases from the RNA-targeted drug discovery field demonstrates the utility of small molecule inhibitors in targeting specific miRNAs (SMIRs) for disease treatment [54]. It has been estimated that the quantity of RNA targets is roughly of at least two orders of magnitude as that of protein targets [55]. In contrast to the growing number of experimentally resolved protein structures in the protein data bank (PDB), the accumulation of RNA-only structures has remained stagnant, with an annual of only 70–100 annually deposited structures over the past decade (∼1% compared to proteins [56]). Moreover, it is likely that miRNAs only make up a small proportion of these structures.

**Fig. 2:**
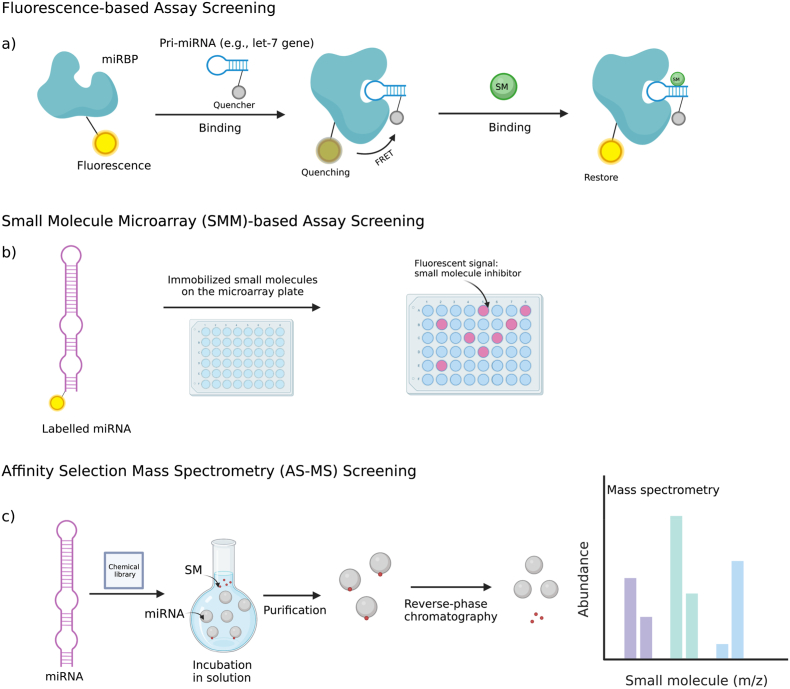
Illustration of chemical approaches for target-centric screening of small molecules in a high-throughput manner. (a) Fluorescence resonance energy transfer (FRET)-based screening. The experiment parameters of this approach are set in a way that a miRNA-binding protein (miRBP, e.g., Lin28A and Lin28B [16]) is tagged with a fluorescence indicator and a quencher is attached to a primary miRNA (pri-miRNA) encoded by the let-7 gene. The binding of the miRBP to the pri-miRNA will induce quenching of the fluorescence. A small molecule inhibitor of the pri-miRNA will restore the fluorescence through disruption of the binding [16,58]. (b) small molecule microarray (SMM)-based assay screening. Small molecules are immobilized on the microarray plate to await the binding of a labelled miRNA [15,59]. Then, high-fidelity signals are shown if the binding occurs. (c) Affinity selection mass spectrometry (AS-MS) screening. Comparatively, the AS-MS-based approach uses label-free targets to screen small molecule inhibitors. The miRNA target is incubated for binding to small molecules. Their complexes are first purified using the chromatography technique and then small molecules are dissociated from the miRNA target using the reverse-phase chromatography technique. Finally, the abundance of different small molecules that can bind to the target is generated using AS-MS. This review collects abundant case studies by using these varying HTS techniques [259].

In addition to high-fidelity structure models, there are several high-throughput screening (HTS) techniques available to precisely and promptly determine SMIRs from a large compound library, to identify lead compounds targeting specific miRNAs involved in human disease pathogenesis [57]. These established techniques mainly include, fluorescence-based assay screening, small molecule microarray (SMM)-based assay screening, fragment-based screening, and affinity selection mass spectrometry (AS-MS) screening [4,8,15,17,51]. These methods are destined for high-throughput screening of large small molecule libraries against one or more specific miRNA target(s), as shown in Fig. 2. Fluorescence-based assay screening approaches possess several derivatives depending on detecting the intensity from fluorescence molecules collectively or individually [58]. In particular, an example of such a derivative is the fluorescence resonance energy transfer (FRET)-based screening approach, which relies on the principle that the fluorescence quenching will not occur if a small molecule inhibitor successfully targets a quencher-attached miRNA that has physically bound to a fluorescence-labelled molecule [16,58]. We only summarized FRET-based screening approach in this review. Small molecule inhibitors are identified by the SMM-based approach in a way that high-fidelity signals are shown on the microarray, in which a library of small molecules are immobilized to attempt to bind to labelled miRNA targets [15,59]. The fragment-based approach leverages a library of small molecules of low molecular weight as fragments to vet the possibilities of them binding to miRNA targets. Then, these possibilities are translated into signals by using, for example, NMR spectroscopy, which are partitioned into two groups to be indicative of bound and unbound fragments, respectively (as depicted in Ref. [60]). Comparatively, the AS-MS-based approach uses label-free targets to screen small molecule inhibitors. Using automated ligand identification system (ALIS), a type of the AS-MS-based approach, binding small molecule ligands are first dissociated from chromatography-purified ligand-target complexes after incubation and then examined from mass spectrometry signals. In addition, there are also a few other approaches for this purpose, such as phenotype-based screening [20], DNA-encoded compound library-based screening [15], and pharmacological validation screening [61]. It is worth mentioning that the phenotype-based approach can work by overlooking the reliance on the knowledge about disease-associated targets and shortlists small molecules that are able to counteract the pathogenic effect of a disease-associated phenotype [57]. The main characteristics of the approaches are summarized in Table 1.

**Table 1:** Summary of important characteristics of several mainstream HTS methods.

HTS Method	Material	Note
Fluorescence resonance energy transfer (FRET)-based screening	Quencher and fluorescence	The binding of a small molecule inhibitor to the pri-miRNA prevents quenching.
Small molecule microarray (SMM)-based assay screening	Immobilized small molecules, microarray plates, fluorescence microscopy	Fluorescence intensities that appear and deepen in a pore on the microarray indicates the binding of a small molecule inhibitor to the miRNA target.
Affinity selection mass spectrometry (AS-MS) screening	Chemical libraries, chromatography, reverse-phase chromatography, mass spectrometry	Purification of compound-binding miRNAs as many as possible is necessary for accurate quantification.
Fragment-based screening	Small molecule libraries, NMR spectroscopy	A library of small molecules of low molecular weight as fragments to vet their possibilities of binding to miRNA targets.

## 5 Clinical applications of ncRNA drugs or targets

Despite the tremendous efforts made to study small molecule inhibitors targeting ncRNAs, clinical applications are still in their infancy [40]. However, miRNAs play an important role in being targeted by drugs or targeting other disease-associated genes [62]. According to the statistics presented by Zhang et al. [63], there are tens of siRNA (as drugs)- and miRNA (as targets)-related therapeutics that have been entered into different phases of clinical trials. For example, RG-101, developed by Regulus Therapeutics, is an GalNAc-conjugated anti-miRNA oligonucleotide sequence to target miRNA-122 against hepatitis C virus infection [40]. However, small molecule inhibitors of miRNAs remain to be tested out only in the lab. For example, using a small-molecule microarray platform called AbsorbArray, a couple of topoisomerase inhibitors were shown to bind to the A bulge in the Dicer site of pre-miR-21 for inhibition of miR-21 expression, which affected the expression of their regulated genes in triple negative breast cancer [64].

In addition, lipid nanoparticle (LNP) drug delivery systems loading with siRNA-based drugs (trade name: Onpattro) has been approved by FDA to treat polyneuropathies [65]. Like the low success rate of discovering drugs for targeting other disease-related agents (i.e., proteins), the commercial development of miRNA therapeutics is confronted with a high failure rate, with a large majority of the therapeutics suspended over the course of drug discovery. But the analysis from Ref. [63] also demonstrates high rates of ncRNA therapeutics (40% for both miRNAs and siRNAs) that are being subjected into phase II clinical trials or more. Nevertheless, generating miRNA therapeutics for disease treatment is still of prime interest to a number of pharmaceutical industries, including miRagen Therapeutics and Regulus Therapeutics [66,67].

## 6 Experimental determination of drug-target interactions

In this review we refer to the interactions between drugs and protein targets as drug-target interactions (DTIs). Mass spectrometry (MS)-based chemical proteomics approaches, including activity-based protein profiling (ABPP) [68] and compound-centric chemical proteomics (CCCP) [69], have been introduced as a powerful tool in high-throughput to yield a large number of interactions between small molecule drugs and protein targets [70,71]. In addition, the evidence for biophysical binding and structural details can also be detected through functionally active protein-compound complexes by the nuclear magnetic resonance (NMR) spectroscopy [72]. The modulation of protein functions is achieved by targeting the protein-protein interaction (PPI) interfaces by chemical compounds [[73], [74], [75]]. As highlighted in Refs. [76,77], NMR excels at capturing the fine-grained local chemical environment/structure compared to cryo-electron microscopy (cryo-EM) [78] as well as X-ray crystallography [79], which makes it more capable of determining DTIs. It is certain that there exist alternative methods for biophysical interaction detection (see this review [80]). There is no doubt that membrane proteins constitute a predominant proportion of all targets [81,82], since they are estimated to involve the transduction of around 85% cell signals [83]. Based on the most recent DrugBank database (version: 5.1.9) [84], we calculate around 50% of all targets as transmembrane proteins, which are targeted by FDA-approved drugs.

## 7 Connectivity scores for drug repositioning

The treatment of cells with drugs can induce changes in gene expression levels, leading to a variety of gene expression signatures (a gene or a group of genes, cf. deregulated genes [85] and drug perturbation signatures [86]) that are differentially expressed as a result of cellular responses to this treatment [87]. Such drug-induced gene expression signatures are conducive to decipher a certain gene expression pattern(s) occurring in a disease and further favour the drug development for the disease [29]. This has given rise to large-scale clinical pharmacogenomic studies [88,89], which yield massive datasets of gene expression matrices induced by a large quantity of drugs, termed drug perturbation datasets [90]. In comparison, there are also drug sensitivity datasets for screening cancer cell lines (see Refs. [31,91,92]). Note that main approaches for miRNA expression profile measurement comprise qRT-PCR, microarrays, and RNA-seq (for details, see Ref. [93]). These pharmacogenomic datasets can be experimentally generated through many cancer models [94], such as patient-derived cancer cell lines [95], organoids [96], and xenografts (PDXs) [97], which serve as valuable resources to predict drug responses and identify biomarkers. We focus on the drug perturbation datasets in the following parts. Despite rapid growth in the number of online resources that are powerful for studying drug perturbation behaviours (e.g., CMAP [29] and a scale-up version of CMAP, L1000 [98], see also a review [30]), there might exist some concerns in terms of the size of the data as well as the quality of the data, as pointed out by Sharifi-Noghabi et al. [91] and Keenan et al. [99], respectively. Nevertheless, by exploiting drug perturbation datasets in conjunction with disease-gene datasets, the associations between drugs and diseases can be established.

**Fig. 3:**
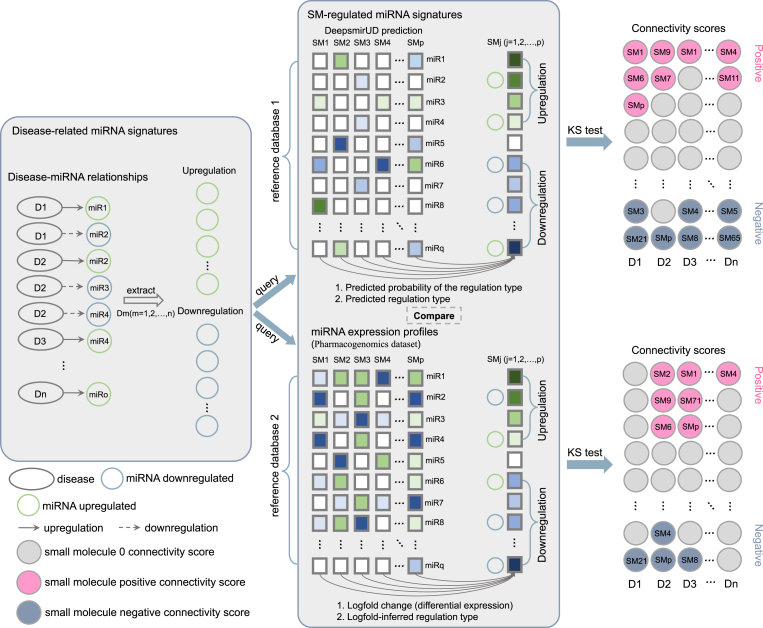
Drug repositioning using connectivity scores. miRNA signatures of a disease of interest are scanned from a database of disease-miRNA relationships and represented by two groups of miRNAs: upregulation or downregulation. Conventionally, the drug repositioning process then queries a reference database for pattern matching of small molecule-induced miRNA signatures with the disease-related miRNA signatures. Such a reference database is obtained from purpose-built large-scale clinical pharmacogenomic studies and represented by a small molecule-induced gene expression matrix. Within each small molecule, the gene signature is generated and ranked by differential expression analysis. The symbols of the resulting logfold change values indicate whether genes within the small molecule are downregulated or upregulated. The ranks of genes within the small molecules are decided by the logfold change values (see Ref. [28] for explanation). Due to the time-consuming nature of the experiment process, the gene expression profiles cannot be accessed easily. Our piloted scheme allows for generating the regulation effect of each small molecule on the expression of each miRNA through only their biological sequences. In this scheme, DeepsmirUD-predicted regulation profiles are used to replace the small molecule-induced gene expression profiles. In comparison, genes within each small molecule are identified as being downregulated or upregulated by the predicted regulation type while their ranks within the small molecule are decided by the predicted probabilities. The final connectivity scores are generated by the Kolmogorov-Smirnov (KS) statistic test to indicate the druglike potential of small molecules using the two routes, respectively.

Connectivity scores have been developed to infer such associations between drugs and diseases. The recently developed recommended connectivity-map scoring method (RCSM) package serves as a repertoire of connectivity scores [28], which contains, for example, the gene set enrichment analysis (GSEA) method and the reverse gene expression score. In the RCSM package, the KS module ranks the gene expression signature of a drug based on the log fold change of gene expression in a bidirectional fashion. In short, this process initiates the classification of the downregulated and upregulated genes of a gene expression signature, and moves to rank these genes within each category, which are however retained in the same gene list where the upmost gene is the one with the biggest log fold change value and the downmost gene is the one with the smallest log fold change value. However, differentially expressed genes (DEGs) identified via a threshold of log-fold change values together with the p-value information based on gene expression matrices (especially miRNA expression matrices) may not easily be accessible due to many ad hoc experimental efforts. Rather than using the information about DEGs derived from expression matrices, the drug repositioning process presented on the DeepsmirUD-Web website exploits a new miRNA expression regulation profile predicted by DeepsmirUD (a recently released deep learning implementation) [27], which we referred to as small molecule-mediated regulatory effects on miRNA expression in the DeepsmirUD work. DeepsmirUD performs the inference of the small molecule-mediated downregulation and upregulation profiles of miRNA expression based solely on molecular sequences of miRNAs and small molecules without relying on an expression matrix specific to a list of drugs in the treatment of disease in a certain cell state. Similarly, this miRNA expression dysregulation profile allows the classification of downregulated and upregulated miRNAs. But differently, it cannot be used to indicate DEGs based on statistical tests because the predicted value for each miRNA represents how likely the miRNA expression is downregulated or upregulated by a query small molecule. Correspondingly, the most top-ranked miRNA represents the one that is most strongly inferred as being upregulated by the query small molecule. We stress this difference in terms of the utilization of statistical tests for examination of the extent of the differential expression genes profile of a gene signature, which makes this scheme a trade-off for drug repositioning based on molecular sequences and connectivity scores. Nevertheless, the DeepsmirUD-predicted downregulation and upregulation profiles make it possible to infer drug-disease associations by virtue of only sequences. These results may be useful to some degree, insofar as we presented a few successfully inferred examples in the DeepsmirUD work [27]. The comparison between the standard scheme and our piloted scheme is schematically illustrated in Fig. 3. We detail the formulation of this drug-disease association process involving DeepsmirUD predictions as follows.

**Fig. 4:**
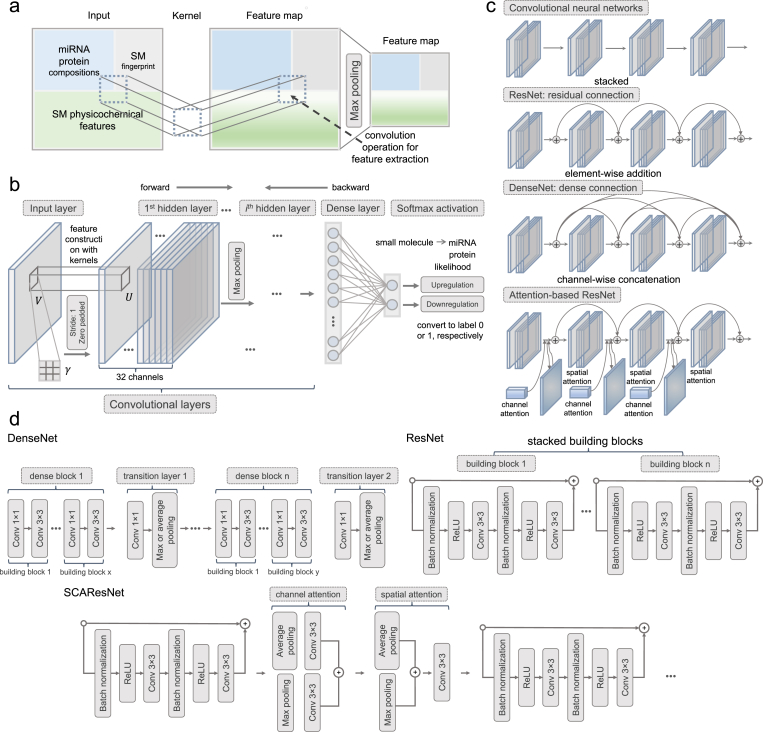
Deep learning approaches for learning and discovering the pattern of relationships (i.e., regulation type or interaction) between small molecules and miRNAs/proteins. (a) Convolution-based deep learning methods extract small molecule and miRNA features. Using the convolution techniques, their initially concatenated feature representations are progressively fused together in their feature maps at high-order neural layers. For example, the boundaries that initially demarcated by different types of features blur as a result of filtering with convolution and subsampling using max pooling. (b) A typical convolutional neural network (CNN) showing a stack of convolutional neural layers placed with multiple filters/kernels for feature extraction over initial input image-like objects of biological sequence features or feature maps, which, after going through a densely connected layer, are processed into highly condensed values to be indicative of relationships between small molecules and miRNAs/proteins. (c) shows four different ways to connect between every two bundles of convolutional neural layers for a CNN (serially connected), a ResNet (residually connected), a DenseNet (densely connected), and an attention-based ResNet (connected with spatial and channel attention modules). (d) shows the detailed building blocks of DenseNet, ResNet, and SCAResNet.

The crux of this idea for drug discovery is the leveraging of statistical tests of genes that are overlapped between the drug-induced gene signature and a list of disease-associated genes [100,101]. One of the earliest connectivity scoring schemes is a type of gene set enrichment score (GSES) calculated based on the non-parametric Kolmogorov-Smirnov (KS) test method [29,102,103], which is integrated into the CMAP 1.0 database [28]. We dubbed this method GSES-KS in this review. Because of its use without relying on an ordered list of disease-associated genes, we have previously introduced this GSES-KS method into the DeepsmirUD work to discover drugs using an unordered list of disease-associated miRNAs from miRCancer [104]. Two KS statistics are first constructed as

$$a=\max_{0\leq i\leq N_{miR}}\left(\frac{i}{N_{miR}}-\frac{p}{L_{SM-miR}}\right)$$

$$b=\max_{0\leq i\leq N_{miR}}\left(\frac{p}{L_{SM-miR}}-\frac{i-1}{N_{miR}}\right)$$

where the miRNA expression signature of drug *A* is denoted as $L_{SM-miR}$ that contains $N_{sm-miR}$ miRNAs and disease-associated miRNA list is denoted as $L_{dz-miR}$ that contains $N_{dz-miR}$ miRNAs. The number of miRNAs that are overlapped between $L_{SM-miR}$ and $L_{dz-miR}$ is $N_{miR}$. p represents the position $i^{th}$ of a gene in the ranked $L_{SM-miR}$ miRNA expression signature in ascending order of the DeepsmirUD-predicted values in both downregulation and upregulation directions.

$$\left\{ \begin{array}{c} {GSES-KS}^{+}=a,a>b\\ {GSES-KS}^{-}=-b,a<b \end{array}\right.$$

where the ${GSES-KS}^{+}$ score indicates a profile for upregulated miRNAs in a given disease and the ${GSES-KS}^{-}$ score indicates a profile for downregulated miRNAs in the disease. Then, the connectivity score GSES−KS is given by

$$GSES-KS=\left\{ \begin{array}{c} {GSES-KS}^{+}-{GSES-KS}^{-},c\\ 0,otherwise \end{array}\right.$$

where c means that both ${GSES-KS}^{+}$ and ${GSES-KS}^{-}$ are positive or negative values. Finally, the connectivity score is normalized as ${\left(GSES-KS\right)}_{norm}$ ranging from −1 to 1. It should be noted that the availability of multiple connectivity scores actually allows much flexibility in building the relationships between the DeepsmirUD-predicted miRNA expression signature and the disease-associated miRNAs. In effect, the application of the connectivity scoring schemes or the CMAP databases has begun to extend to a few fields, including identifying active ingredients from traditional Chinese medicine [105,106].

## 8 Reverse pharmacological profiles of structurally similar small molecules

It is crucial to exercise caution when processing drug-target association/interaction data, as it may significantly impact the quality of computational analysis and prediction. Small changes in the structure of a molecule can lead to large perturbations in its chemical activity, a phenomenon that is well known in molecular design [[107], [108], [109]]. It has been suggested that a slight chemical modification on an antagonist, which binds to the human A3 adenosine receptor (hA^3^AR), turns it to an agonist [110]. In the hA^3^AR-agonist model, the agonistic compound induces an intense conformational change of the hA^3^AR, while in the hA^3^AR-antagonist model, the antagonistic compound keeps the hA^3^AR activity in check [110,111]. By comparing the two structures, we find that two compounds derived from their respective models are shown to be highly structurally similar, as evidenced by a Tanimoto coefficient of 0.992. Both agonistic and antagonistic modes of this compound are of pharmaceutical significance to act on the pathogenic mechanisms of different diseases as potential drugs. However, it poses a grand challenge to computational analysis. For instance, if the two triplets of drug-target responses (hA^3^AR-agonist-activation and hA^3^AR-antagonist-inhibition) are catalogued into a cohort for a two-label classification problem, the optimization process or the ultimate performance of machine learning models could deteriorate by simultaneously considering the two samples with extremely similar features (i.e., hA^3^AR-agonist) but completely different labels (i.e., activation and inhibition). This is because the association types (labels) that are formed biologically are sometimes independent of the sequence or structural similarity between small molecules, miRNAs, or both. Therefore, this could potentially complicate the situation of reducing the redundancy of drug and target data, i.e., particularly whether to remove those similar samples across all categories (activation and inhibition) or simply within each category.

## 9 Deep learning

### 9.1 Convolution-based deep learning architectures

Deep learning algorithms are methods that learn representations by automatically extracting features from input data [112]. This enables a diminished involvement of domain experts with the preparation of crafted features, which makes it possible to gain a high degree of accuracy that is equivalent to the utilization of features from a tedious, arduous preparation process [113]. The learning process involves linear (e.g., the addition operation) or non-linear (e.g., the ReLU activation function [114]) transformations of input data being fed into neurons from one to another neural layer [115]. To satisfy these meets, deep learning algorithms often have proprietary architectures, which are constructed using a few basic unit layers, including convolutional layers [116], recurrent layers [117], and graphical convolutional layers [118]. As summarized in this review [119], the same or different types of these layers are assembled into convolutional neural networks (CNNs) [120], recurrent neural networks (RNNs) [121], and graphical convolutional neural networks (GCNs) [122] through, for example, parameter sharing settings [123]. For deep neural networks pertaining to convolutional layers, subsampling is inevitably involved in their assembly processes as it can reduce computational costs significantly [124]. Common subsampling strategies include channel-wise or element-wise max-pooling or average-pooling operations [125].

We have found the use of deep learning architectures of convolutional layers connected in multiple ways to be highly effective in predicting small-molecule-mediated regulatory effects on miRNA expression [27] as well as drug-target interactions (given in sections below). Therefore, we mainly confine the deep learning part of this review to the introduction of the convolution-based models. We present a couple of representative architectures, including ResNets [126], DenseNets [127], and SCAResNets [128], that produce a series of stably varying values of evaluation metrics (e.g., AUC or AUCPR) across training epochs than other types of architectures in the two learning tasks above. The main characteristics of the models used in the DeepsmirUD work are summarized in Table 2. The similarity and difference between these models are summarized as follows.

1. **Similarity.** Apart from taking all convolutional layers as its backbone structure, all these models culminate with a fully-connected layer for converting all previously learned features into a possiblity for indicating a binary label (e.g., upregulation or downregulation). These models are structured as a connection of building blocks, each alternating between batch normalisation, non-linear activation functions, and/or convolutional layers in a certain order (see Table 3). For example, for the pre-activation design of a building block, batch normalisation is placed first, which is followed by the ReLU actvition function and a convolutional layer. All the three types of models utilize average pooling or max pooling for subsampling to reduce the size of feature maps.
2. **Difference.** The convolution-based deep learning models differ primarily in the way how building blocks connect to each other and the time required for training.
• *Building block connection.* As shown in Fig. 4c, there are four ways to connect between convolutional layers**.** The raw CNNs are a stack of serially connected convolutional layers without applying additional operations [129]. In a residual neural network, every two convolutional layers are connected in a residual manner as a residual unit in which the final output of it is the sum of the output from the second convolutional layer and the raw input to the first convolutional layer, which is also called the identity shortcut (see Table 3). The invention of ResNets is conceived as a milestone in the development of deep learning algorithms, since this type of network is widely believed to allow for an ultrahigh speed to train an ultradeep CNN with the layer number equal to it. Inspired by the residual connection scheme, Huang et al. designed an architecture of densely connected layers, termed DenseNets, where convolutional blocks, each represented by a few serially connected convolutional layers, are connected in a way that the output of all convolutional blocks is the sum of the output of the last convolutional block and the sum of the output of all the preceding convolutional blocks [130]. The main difference between the two types of connections is that DenseNets allow for reusing the extracted features from all previous building blocks while ResNets can only use the feature information from its preceding one building block. The performance of DenseNets was tested to be superior to that of ResNets. Another study employed an attention-based mechanism [131] to further enhance the prediction performance using ResNets [192]. The approach incorporated spatial and channel-wise attention (SCA) modules onto the initial output of every residual unit. These SCA modules, together with the raw input to that residual unit, are then added up to the input to the next residual unit. Essentially, the SCA modules reinforce feature extraction from specific regions of feature maps by introducing two extra sets of weights in addition to the weights of the kernels in each residual unit. A more comprehensive explanation can be found in Ref. [132].
• *Time and space complexity.* The development of new deep learning architectures for image recognition has been focused on shortening their training time at no cost of prediction accuracy or at even improved accuracy [133]. Since the parameters of those architectures involving convolution operations are sourced solely from filters, the size and number of filters are of direct relevance to the issue on computational costs. Theoretically, the time complexity of executing a single convolution operation in a convolutional layer is kept unchanged if the input and the filter are set as the same in that layer, regardless of whatever types different deep learning architectures are. However, there can be much pronounced difference between deep learning architectures in terms of the space complexity. For example, DenseNets consume much more Ram/GPU memory than ResNets to handle more complex concatenation operations between building blocks. It is reported that compared to ResNets, the use of DenseNets leads to a 1.2-fold increase in the training time [134]. Since SCAResNets are constructed with additional attention modules (i.e., additional convolution) on top of ResNets, they are doomed to require longer time for model training than ResNets.

**Table 2:** Deep learning models adopted in the DeepsmirUD work.

Model	Conv layer number	BB number	Conv 1 × 1 number	Conv 3 × 3 number
CNNs	3	1 (1)	–	3
ResNet18	17	8 (2, 2, 2, 2)	–	16
ResNet50	19	16 (3, 4, 6, 3)	32	16
DenseNet41	40	18 (6, 3, 3, 6)	21	18
SCAResNet18	17	8 (2, 2, 2, 2)	–	16

**Note:** Conv: convolutional. BB: building block. Conv 1 × 1 is used for setting a bottleneck architecture that can significantly reduce the number of parameters in filters and yet keep the dimension of feature maps unchanged [126]. Each number separated by a comma in brackets represent the number of basic building blocks in each stacked building block structure (see also Fig. 4d).

**Table 3:** Examples of convolution-based deep learning architectures.

Architecture	Structure within a BB	Connection between BBs	Subsampling	Attention mechanism
CNNs	–	–	max pooling	No
ResNets	conv layers, ReLU, BN	identity shortcut	max/average pooling	No
DenseNets	conv layers	dense connection	max/average pooling	No
SCAResNets	conv layers, ReLU, BN	identity shortcut	max/average pooling	Yes

**Note**: Conv: convolutional. BB: building block.

### 9.2 Weight determination using the backpropagation algorithm

The above derivatives of CNNs are built by hinging on a basic structure, namely, the convolutional layer in which kernels (also called filters containing weights or parameters) are placed to perform convolutional operations on image objects along horizontally and vertically. We next set out to mathematically formulate this process. To made it clear, we show in Fig. 4b that a kernel between two adjacent layers contains the weight matrix Υ that is required to be determined, which can be achieved by the backpropagation algorithm [135,136]. CNNs are feedforward neural networks through which information is propagated along the forward- and backward-pass directions [137,138], as indicated by upper arrows in Fig. 4b. During a forward pass, the information flows from a feature map in layer l to another feature map in layer l+1 [139], which is written as

$$u_{m,n}^{l+1,d}=\sum_{d=0}^{D}\sum_{p=0}^{P}\sum_{q=0}^{Q}\gamma_{p,q}^{l+1,k,d}\times v_{m+p,n+q}^{l,d}$$

where D is said to be the number of channels, and P and Q are the length and the width of a kernel, respectively. $\gamma_{m,n}^{l+1,k,d}$ represents the weight at row p and column q in the weight matrix Υ (i.e., the k th kernel) at the dth channel in layer l+1. $v_{m+p,n+q}^{l,d}$ represents the element (i.e., a small molecule or a miRNA feature as in the DeepsmirUD work) at row m+p and column n+q in the feature map V at the dth channel in layer l. $u_{m,n}^{l+1,d}$ represents the element at row m and column n in the output U at the dth channel in layer l+1. It can also be rephrased to its matrix form, such that,

$$U^{l+1,d}=\sum_{d=0}^{D}\Upsilon^{l+1,k,d}⊗V^{l,d}$$

where ⊗ represents the convolution operation. Let J be the cost function. During the backward-pass process, in each epoch, the weight $\gamma_{p,q}^{l,k,d}$ at row p and column q in the weight matrix Υ at the dth channel in layer l+1 is updated by the gradient descent method [140,141].

$$\gamma_{p,q}^{l+1,k,d}=\gamma_{p,q}^{l+1,k,d}-\alpha \frac{\partial J}{\partial \gamma_{p,q}^{l+1,k,d}}$$

$$\frac{\partial J}{\partial \gamma_{p,q}^{l+1,k,d}}=\sum_{m=0}^{M-P}\sum_{n=0}^{N-Q}\frac{\partial u_{m,n}^{l+1,k}}{\partial \gamma_{p,q}^{l+1,k,d}}\times \frac{\partial J}{\partial u_{m,n}^{l+1,k}}$$

$$\frac{\partial J}{\partial \gamma_{p,q}^{l+1,k,d}}=\sum_{m=0}^{M-P}\sum_{n=0}^{N-Q}\frac{\partial J}{\partial u_{m,n}^{l+1}}\times v_{m+p,n+q}^{l,d}$$

$$\frac{\partial J}{\partial \Upsilon^{l+1,k,d}}=\frac{\partial J}{\partial U^{l+1,k}}⊗V^{l,d}$$

where M and N represent the length and the width of an input feature map. $\frac{\partial J}{\partial U^{l+1,k}}$ is calculated based on its counterpart in layer l+2, as demonstrated in Ref. [141]. The loss, which is the difference between predicted labels (i.e., 0 or 1 converted after softmax activation in Fig. 4b) and ground-truth labels, can be calculated after all weights are updated in each epoch. In summary, weights in matrix Υ for any kernel can be updated iteratively according to above equations and stop updating until the loss between two epochs changes in a meagre range.

## 10 Computational prediction of small molecule inhibitors

In this section, we introduce the computational techniques for discovering small molecules inhibitors of miRNA and protein targets, which includes RNA motif-searching prediction, association prediction of small molecules and miRNAs (SM-miRs), regulation type prediction of associated SM-miRs, and drug target interaction (DTI) prediction. Most of the prediction processes require the expansion of data as input by indicating unknown SM-miR or drug-target relationships and deep learning modelling needs a feature engineering process, which are shown in sections 10, 10.1.1.1.2, respectively.

### 10.1 Data augmentation and feature preparation

#### 10.1.1 The guilt-by-association rule

Biological systems are often described as complex networks [142,143], in which biological entities such as small molecules, proteins, and genes are represented as nodes and the relationships between them are represented as edges. These biological networks can be further categorized into homogeneous and heterogeneous networks [144]. In detail, biological entities of one kind (e.g., drugs) are connected with one another into the homogeneous network, such as the drug-drug interaction network, while biological entities of different kinds (e.g., drugs and targets) are inter-connected into the heterogeneous network in which its two representations, bipartite [145] (e.g., the drug-target interaction network) and tripartite [146] (e.g., the drug-target-disease association network or the drug-target-response network) graphs, are very commonly seen in drug discovery fields [[147], [148], [149]]. While heterogeneous networks usually contain a hefty volume of nodes, the relationships between these heterogeneous nodes can largely be unknown [146,150]. One approach for deducing such unknown relationships is the guilt-by-association (GBA) approach. It operates under the assumption that if biological entities with unknown function have an interaction profile similar to those of known function, they may function similarly [[151], [152], [153]]. This implies that if drug A is known to treat a disease, the GBA-deduced drugs similar to drug A may also potentially be used to treat this disease. This approach has been demonstrated very useful in drug discovery [146,150,154,155]. Often, the relationships in heterogeneous networks are deduced by utilizing information from a medley of homogeneous networks [144]. For example, if drug A is linked to protein X in a drug-protein interaction network, a new link between protein X and drug B in this network is required to be built by comparing the interaction profile between drug A and drug B in an external drug-drug interaction network where the two drugs coexist. Similarly, finding new protein targets of a drug in this drug-protein interaction network requires the information from an external protein-protein interaction network. The drug-drug interaction network or the protein-protein interaction network can be established by using those metrics that reflect its sequence, structural, functional properties, such as Tanimoto coefficients (for evaluating the chemical similarity between drugs) [156], sequence identity (for evaluating the sequence similarity between proteins) [27], or the direct experimental evidence to support interactions between drugs or proteins [157].

#### 10.1.2 Feature engineering for ribonucleic acids, amino acids, and compounds

Computational studies of SM-miRNA interactions and DTIs often require a great amount of feature engineering work to characterize the biophysical and biochemical properties of miRNAs, proteins, and compounds. Compositions of miRNAs and proteins are well-suited for machine learning approaches taking the entire molecular sequences as input, since this kind of feature can yield a fixed-length feature encoding vector regardless of the issue on the input sequences of varying lengths. The compositional features are, in short, the frequencies of k-mers, where k usually takes 1, 2, or 3 at better, leading to 20, 400, 8000 dimensions for proteins for example. Tools that are powerful for generating these features include propy [158], PyDPI [159], PyBioMed [160], and PyFeat [161] available on Python interfaces, and iFeature [162], iLearn [163], iLearnPlus [164], and iFeatureOmega [165] available on webserver interfaces. In contrast, compounds have more complex features suitable for machine learning modelling, for example, fingerprints for compound's structural representations or partition coefficient (log P) and molar refractivity [166] for compound's physicochemical properties. There is, perhaps, no caveat to utilize diverse compound features for interaction prediction problems. The RDKit tool is widely applied in this aspect [167].

### 10.2 Computational design of lead compounds based on miRNA motifs

Inforna is a web server that provides computational design of lead small molecule compounds that target RNA molecules. It works mainly as an algorithm-embedded, expert-curated database of RNA motif–small molecule relationships [21]. With only an input RNA sequence of any length, Inforna undergoes the two stages of prediction-based comparisons: predicting its secondary structures and predicting the fitness of an RNA motif–small molecule interaction through the StARTS scoring scheme to produce lead compounds [22]. The concept is that if the predicted secondary structures of the input RNA share a high similarity with the RNA motif paired with a small molecule, the input RNA can be linked to that small molecule [168]. These deposited RNA motif–small molecule relationships are rendered reliable since they are derived from high-quality microarray-based screening experiments [169]. Several experiments provide proof of the accuracy of computation-led selection of lead compounds [170,171]. For example, Suresh et al. reported that a compound prioritized by Inforna to target the Dicer processing site in pre-miR-21 resulted in a moderate inhibitory effect in treating triple-negative breast cancer [172]. Later, Liu et al. showed in another experiment that a Inforna-designed compound successfully targeted a motif in the Dicer processing sites of several pri-miR-17-92 family members (miR-17, miR-18a, and miR-20a), with the potential to treat polycystic kidney disease [173].

### 10.3 Prediction of associations between small molecules and miRNAs

A number of models have been proposed to predict the SM-miR associations based on network/matrix-based inference approaches and machine learning techniques. As of now, we have collected from the literature 20 methods, of which 12 are categorized into the first category, including BNNRSMMA [174], EKRRSMMA [175], WKNKN [32], RWNS [176], GISMMA [177], SMANMF [178], SLHGISMMA [179], TLHNSMMA [180], HSSMMA [181], CLDISMMA [182], SMMART [183], RWR [184], and SNMFSMMA [185], and 7 belong to the latter group, including ELDMA [186], PSRR [187], DAESTB [188], RFSMMA [189], SMAJL [190], Abdelbaky et al. work [191], and Jamal et al. work [192]. The categorization is not restricted because some of the network/matrix-based methods, e.g., the SMMART work, may also involve an optimization process yet without a negative training dataset, thereby being excluded from the machine learning category. Some of the methods, e.g., the Abdelbaky et al. work, offer disease inference for outreach to showcase the application of drug discovery for a particular disease. The golden standard database that is most prevalently used among these methods is SM2miR [193], released one decade ago. In an effort to make a fair performance comparison, researchers have created two publicly available datasets of well-curated SM-miR associations, which most studies named Dataset 1 and Dataset 2. But the SM-miR association database is not only confined to SM2miR. For example, Jamal et al. initiated a collection of more than 300 thousand small-molecule modulators and/or inhibitors of miR-21 via the PubChem AID 2289 by using the quantitative HTS (qHTS) assay [192]. Through an activity scoring scheme, these small molecules are tagged as active or inactive, which are then subject to data mugging and feature engineering and finally delivered to the Naïve Bayes [194] and random forest [195] learning algorithms for inferring the inhibition likelihoods of miRNA targets. The SM-miR association prediction field is undergoing rapid progress especially in the past few years, in terms of the surge in the number of method-based publications and the predictive ability. According to the performance comparison in these studies, the two newest methods, WKNKN and DAESTB, improved AUC values from 0.863 to 0.986 on the two datasets. As can be seen above, this field lacks machine learning implementations, especially deep learning implementations. The primary reason is that in order to avoid overfitting brought about by the label imbalance problem [196,197], machine learning algorithms usually seek to be trained on label ratio-balanced samples, but the current SM-miR databases are filled with known association samples instead of non-association samples. What is more important, manually/computationally added putative negative samples (i.e., non-associations) may harm the performance of machine learning models, as discussed in Ref. [183].

In addition to the direct connections between drugs and miRNAs, some studies have incorporated functional data on drugs, such as drug responses, to model their relationship with miRNAs. For instance, Huang et al. developed the GCMDR approach to predict the associations between drug resistance and miRNAs [198], while Li et al. employed a heterogeneous network framework to predict the relationships between anticancer drug responses and miRNAs [199]. Unlike the SM-to-miRNA regulation direction, which involves a direct physical interaction between them, both studies explored how drug responses are modulated by miRNAs. This line of research is referred to as miRNA pharmacogenomics, which aims to understand the mechanism of drug action on diseases by linking drugs, miRNAs, and genes together [200]. Specifically, the rationale is that the response of a drug is affected by miRNAs through the dysregulation of the expression of genes whose protein products bind to this drug. These kinds of triplets (miRNAs- > genes- > drugs), where the drug response lies at the very last link of the chain reaction, indirectly link miRNAs and drugs, as indicated by Rukov et al. [201].

### 10.4 Prediction of regulation type of associated small molecules and miRNAs

Computational approaches have advanced the discovery of drugs targeting miRNAs for disease treatment, following the progress made in predicting SM-miR associations [202]. Summarized in this review [203], drugs can be repositioned efficiently by an *in-silico* gauge of the presence or absence of SM-miR associations. The expression of miRNAs can be altered either upwards or downwards by binding with different small molecules, but currently, this information seems not to be seamlessly integrated with the later computational drug discovery process that combines with the disease knowledge. For example, the pathogenesis of a disease is related to the upregulation of the expression of a miRNA. Then, we pursue whether there are small molecules that are able to downregulate the miRNA expression. Knowing the importance of the SM-miR regulation types to the development of potential small molecule therapeutics [54,61,204,205], we have developed two computational tools, DeepsmirUD and DeepdlncUD (manuscript in preparation), to predict the regulation types (cf. we referred to as regulatory effects in the two studies) of small molecules for altering the expression of miRNAs and lncRNAs, respectively. It is noted that a SM-miR pair taken as input to DeepsmirUD has to be an experimentally-verified or predicted association. After then, with the regulation type-specific information, miRNA expression signatures induced by small molecules were used to couple with disease-related miRNAs to find potential drugs by computing connectivity scores (details summarized early in this review). The data availability is of concern to the computational method development. We downloaded two data resources, SM2miR [193] and D-lnc [206], which made it possible to access the machine learning approaches in miRNAs and lncRNAs, respectively. The regulation type prediction has not reached other types of ncRNAs as we did not collect a hoard of interaction data that suffices to train a machine learning model.

### 10.5 Prediction of interactions between drugs and protein targets (DTIs)

#### 10.5.1 Databases of DTIs

**Fig. 5:**
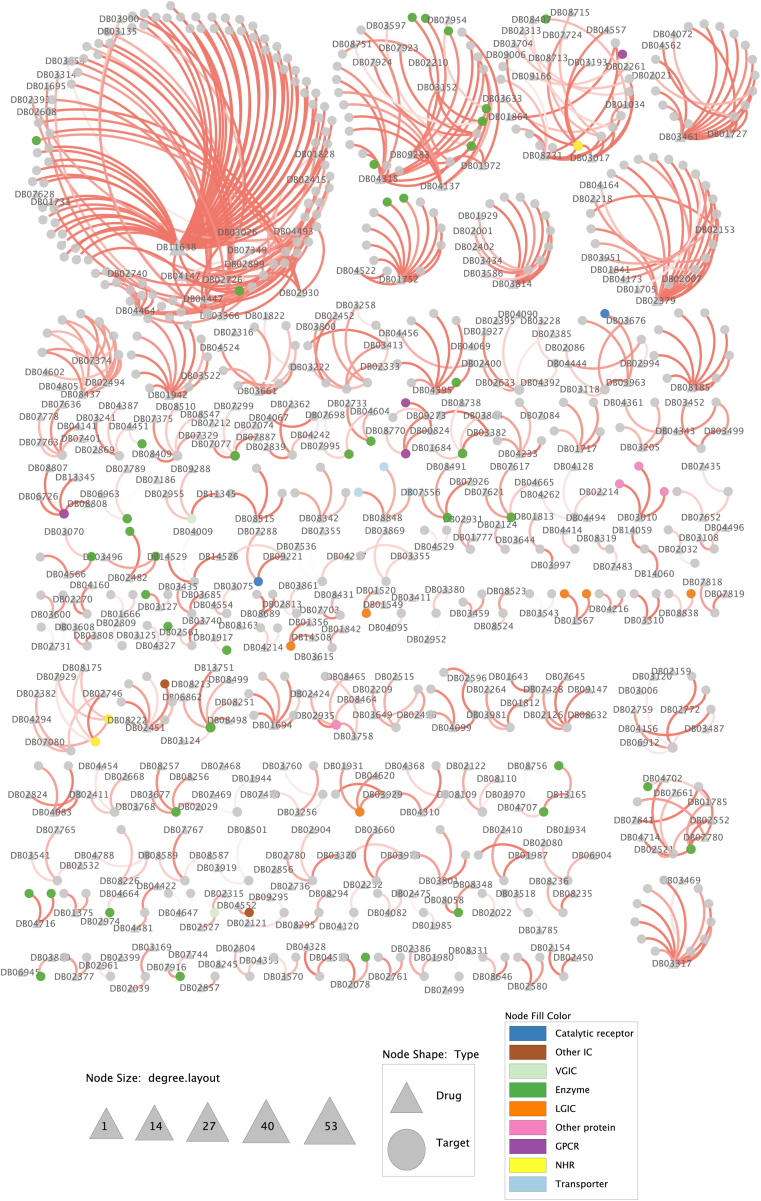
Network of DTIs formed with novel therapeutic targets. Each line represents ground-truth evidence of an interaction between a protein target and a drug. The connection strength of each interaction is indicated by using the Drutai-predicted DTI probability. Drugs and protein targets are represented by the triangle and the circle, respectively. The size of the triangle of a drug becomes bigger if the drug is identified to connect with more targets (see https://aidrugud.github.io/drutai for interactive graphs). Human transmembrane proteins that are of pharmacological significance are annotated based on the GtoPdb database [260] using 9 functional groups: *Transporter*, *GPCR* (G-protein-coupled receptor), *Enzyme*, *Catalytic receptor*, *LGIC* (ligand-gated ion channel), *VGIC* (voltage-gated ion channel), *NHR* (nuclear hormone receptors), *Other IC* (other ion channel), and *Other protein*.

**Fig. 6:**
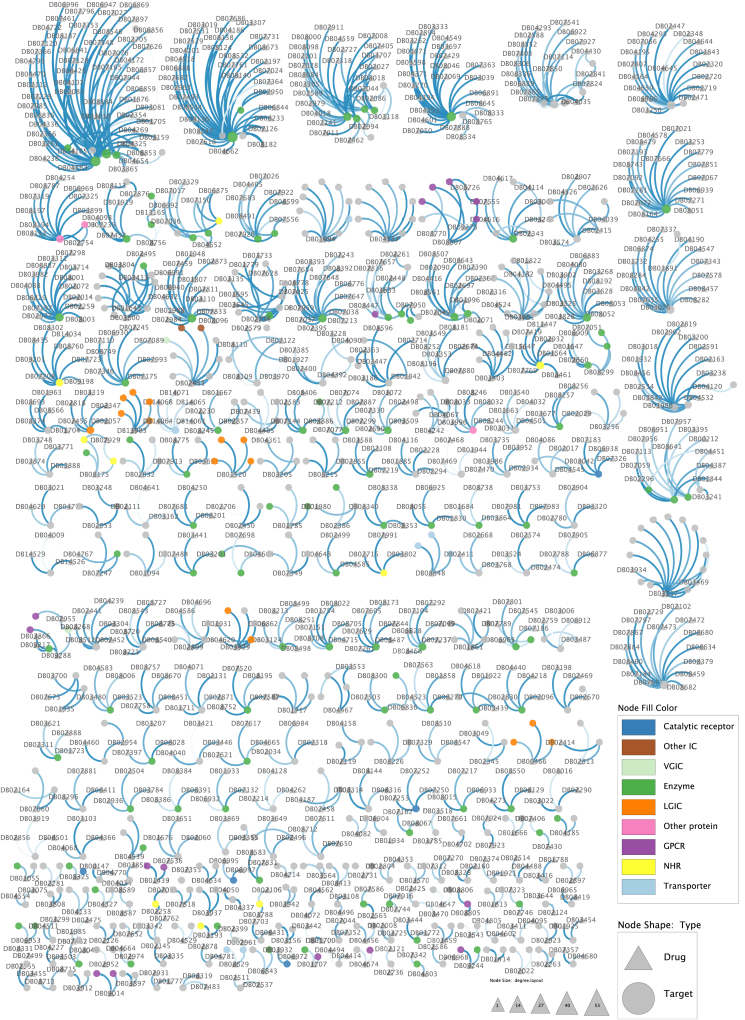
Network of DTIs formed with novel drugs. Each line represents ground-truth evidence of an interaction between a protein target and a drug. The connection strength of each interaction is indicated by using the Drutai-predicted DTI probability. Drugs and protein targets are represented by the triangle and the circle, respectively. The size of the triangle of a drug becomes bigger if the drug is identified to connect with more targets (see https://aidrugud.github.io/drutai for interactive graphs). Human transmembrane proteins that are of pharmacological significance are annotated based on the GtoPdb database [260] using 9 functional groups: *Transporter*, *GPCR* (G-protein-coupled receptor), *Enzyme*, *Catalytic receptor*, *LGIC* (ligand-gated ion channel), *VGIC* (voltage-gated ion channel), *NHR* (nuclear hormone receptors), *Other IC* (other ion channel), and *Other protein*.

As high-throughput experimental techniques are being increasingly applied for screening drug candidates that activate or inhibit protein targets related to a certain disease(s), there have been a growing number of well-established databases of DTIs with a concomitant increase in the number of DTIs [207], including DrugBank [84], TTD [208], SuperTarget [209]. The information about other available webservers managing the DTI data has been well-documented in these three reviews [33,210,211]. We mainly focus on the analysis of the DrugBank database due to its pervasive applications in computational DTI studies. The data is compiled in DrugBank at different levels of granularity, such as SNP-associated drug effects, drug–drug interactions (DDIs), DTIs, etc. Following a major overhaul in its 5th version, DrugBank has substantially expanded its data content, with several categories experiencing at least a 1-fold increase (e.g., 600% for DDIs) and a few recent additions, such as drug clinical trial data. While the precise number of DTIs is not explicitly revealed in this version change, statistics calculated based on the two most recent versions (5.1.8 and 5.1.9 downloaded through the target sequence mark) indicate a slight increase in the number of DTIs, with a 3.13% increase for FDA-approved-drugs and a relatively unchanged ratio for experimental-drugs (i.e., preclinical or animal testing phase) (Table 4). Furthermore, there has been a noteworthy increase of approximately 6.46% in the number of protein targets that have been identified as being targeted by FDA-approved drugs. For example, there are 57 new drugs tagged as FDA-approved. After removing repeated records and those target sequences containing non-standard amino acid symbols, the number of DTIs decreases 0.1-fold for FDA-approved drugs and 0.05-fold for experimental drugs. Overall, the recent DrugBank database maintains ∼10,000 DTIs per each category, which serve as adequate materials for computational modelling and analysis. Based on the DrugBank database, we generated a DTI dataset to train a predictor, Drutai, in order to determine how accurately deep neural networks can predict DTIs using molecular sequences alone (see Supplementary Materials). Then, we use Drutai to predict DTIs when targets and drugs are novel (Fig. 5, Fig. 6, see https://aidrugud.github.io/drutai for interactive graphs), respectively.

**Table 4:** Number of drugs, targets, and DTIs in DrugBank.

Version	Condition	Approved			Experimental		
		Target	Drug	Interactions	Target	Drug	Interactions
5.1.8	raw	2694	2170	11058	2984	4549	8698
	non-repeated non-UNK	2682	2167	10738	2970	4548	8549
	PL < 3000	2484	1839	9776	2851	4481	8306
5.1.9	raw	2868	2227	11415	2983	4551	8692
	non-repeated non-UNK	2856	2224	11083	2969	4550	8543
	PL < 3000	2650	1872	10,087	2837	4482	8280

Note. UNK: unknown characters out of 20 standard amino acid symbols. PL: protein length.

#### 10.5.2 Computational tools for predicting DTIs

The development of therapeutics often involves using druglike small molecules to bind to protein targets for inhibition or activation [212]. Consequently, predicting DTIs has garnered significant attention as it can greatly reduce the experimental efforts required to test the therapeutic potential of small molecules [23]. Since then, improving the prediction of interactions between drugs and protein targets is one of the most promising fields in computational drug discovery [213]. With accumulated computational efforts, this research area has now been overwhelmed by numerous predictors, which are mainly categorized into sequence-based and structure-based predictors. There are too many sequence-based approaches to enumerate, such as Yu et al. work [214], EnsemDT [215], and DTINet [216]. Recent years have also seen a rising number of deep learning methods, many of which conceive novel schemes of taking features of DTIs as input to deep neural network architectures [217,218]. For example, the learning scheme implemented by DeepDTA [219] is that a small molecule and a protein are fed into two separate CNNs to be trained in parallel for learning their latent representations, which are then concatenated into one feature vector that is finally translated by conventionally a fully-connected neural network into the propensity of it being in interaction or non-interaction. Likewise, other methods that adopt a similar idea include DeepAffinity [220], DeepPurpose [221], MDeePred [222], MINN-DTI [223], MultiDTI [217], and HyperAttentionDTI [224]. It is worth mentioning that in the MDeePred, an image-like object that hierarchically represents protein features through channels is used as input to a deep learning architecture, while in the MINN-DTI work, the distance map is leveraged to improve the representation of target proteins. Besides, the utility of graphs involved in the sequence-based prediction has been demonstrated by a few studies [[225], [226], [227]]. The crux of the plan is to represent a chemical structure as a graph or add relevant protein-protein or drug-drug interaction networks, which fit into graph convolutional neural networks (for review, see Ref. [228]). Comparatively, the structure-based prediction has yet to be popularized mainly due to the availability of protein structures, the additional computation on structures, and the lack of the effective plans of the incorporation of protein structures and learning algorithms. Existing studies have given several examples to incorporate protein structures into the prediction DTIs, e.g., protein sequence annotations in domain resolution [229], graph representations of binding pockets [230], and predicted residue contact maps [231]. The DrugBank database [84], as an internationally trusted source of DTIs, is involved in a large part in the development of many methods, such as DrugE-Rank [232], LASSO-DNN [156], DeepDTIs [233], DrugR+ [234]. To illustrate the applicability of the prediction tools, DTIs formed with novel drugs or novel targets are curated as in Refs. [[235], [236], [237]].

### 10.6 Network analysis of the connectivity scoring scheme using drug-mediated miRNA profiles predicted by DeepsmirUD

To illustrate the utility of our piloted scheme based on connectivity scores to reposition conventional drugs or discover new drugs, we carried out a drug-miRNA-disease network analysis. To this end, we extracted from DeepsmirUD-Web [27] the drug-mediated miRNA profiles with absolute values of predicted regulatory effects (i.e., likelihoods of upregulation or downregulation types) of greater than 0.95 and the drug-disease associations with absolute values of connectivity scores of greater than 0.5.

**Fig. 7:**
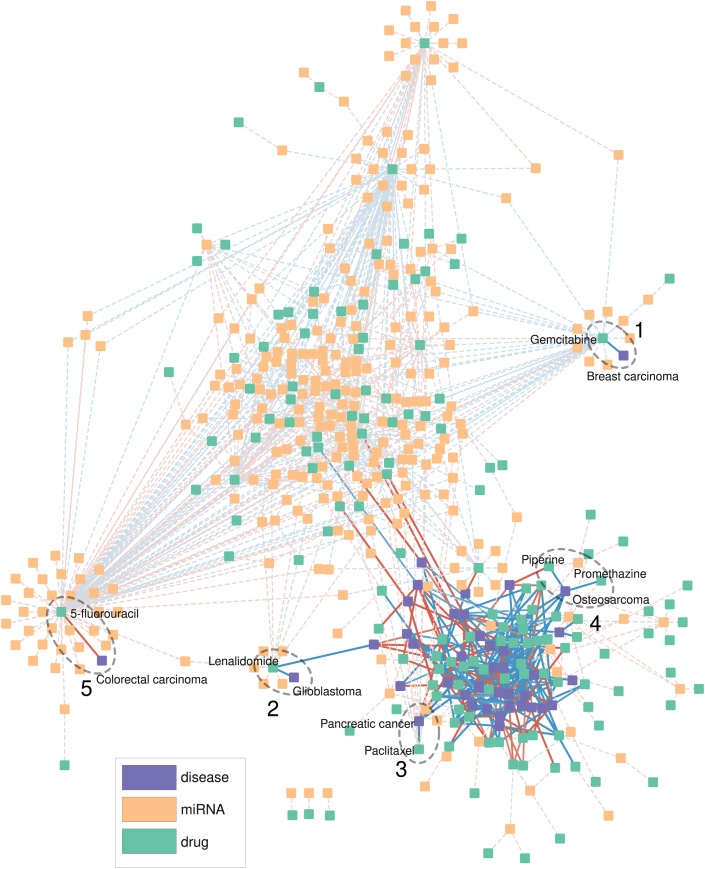
Network analysis based on small molecule-miRNA, miRNA-disease, and small molecule-disease relationships extracted from DeepsmirUD-Web. The five ellipses highlight the predicted druglike potential of five small molecules (gemcitabine, lenalidomide, paclitaxel, piperine, and 5-fluorouracil) for treating breast carcinoma, glioblastoma, pancreatic cancer, osteosarcoma, and colorectal cancer, respectively. The blue line indicates the druglike potential of a small molecule while the red line indicates the ability of a small molecule to be supportive of a disease. The blue and red dashed lines indicate the downregulation and upregulation type of small molecules on modulating miRNA expression, respectively. Small molecule-disease relationships are linked based on connectivity scores [28].

We present five case studies shown in Fig. 7. In ellipses 1–3, our network analysis successfully predicts the capability of gemcitabine [238], lenalidomide [239], and paclitaxel [240] to treat breast carcinoma, glioblastoma, and pancreatic cancer, respectively, as highlighted by the negative weights ranging between −1 and 0. Considering its several side effects, such as nausea, fatigue, low blood cell counts, and allergic reactions [241], gemcitabine should cautiously be used for treating breast carcinoma. However, the network created using the connectivity scoring scheme is also full of wrong indications or links that remain unknown. Apart from a successfully predicted true link between piperine and osteosarcoma [242], ellipse 4 gives a false or currently unknown link between promethazine and osteosarcoma. Moreover, 5-fluorouracil, as a chemotherapy medication, has a potential therapeutic effect on colorectal cancer (CRC) [243]. However, there is the other way around in ellipse 5, indicated by the positively connected strength of 5-fluorouracil with CRC. It is noted that due to its chemoresistance, 5-fluorouracil is suggested to be better used in conjunction with other natural or synthetic compounds for the co-treatment of CRC.

## 11 Discussion

This review provides an overview of miRNA biogenesis, functions, and disease implications, from both biological and computational perspectives. Additionally, it presents the current advancements in computational strategies for the discovery of small molecule drugs of miRNA targets. By focusing on the small molecule-mediated targeting of miRNAs, this review is structured in a concise format to summarize experimental technologies for drug screening in tandem with computational strategies. To link drugs and diseases, we begin by stressing the importance of associations of miRNAs with them, respectively, since aberrant biomolecules (say, genes, transcripts, and proteins) constitute the genetic and phenotypic basis that underlies the pathogenesis of diseases [14], and these molecules provide the structural basis that accounts for exerting cellular functions in somewhat of a high ratio by interacting with small molecule compounds [18]. In particular, compare to targeting proteins, targeting miRNAs with druglike small molecules is detailed by a large margin of this review. This is because the development of miRNA-targeted small molecule inhibitors has burgeoned a new field, where drugs are found through the post-transcriptional regulation mechanism of miRNAs rather than using protein information. This way is more straightforward owing to the miRNAs’ direct regulation control of deregulated gene expression that triggers diseases and, furthermore, the control of protein noises by targeting lowly expressed genes, as evidenced in an experiment on mouse embryonic stem cells [244]. Specifically, we elaborate the crucial role of miRNAs in developing cancer therapeutics from their biogenesis, functions, and experimental determination, eventually to the clinical applications, in that their aberrant expression is thought to affect cell proliferation, differentiation, and apoptosis. miRNA-targeted small molecule inhibitors have been proven to be useful to antagonize oncogenic pathways. Creating a solution for drug discovery is inextricably linked with experiment-led and computation-assisted efforts in concert. Experimentally, the discovery process with current technologies is accompanied by a time-consuming preparation of a bespoke chemical library for high-throughput screening, which makes drug determination challenging. Rather than substituting the existing models, computational techniques should play a supporting yet sometimes dominant role in accelerating the duration of drug discovery. Overall, the clinical development of miRNA drugs is still considerably enroute.

The state-of-the-art computational capabilities have actively been used in the field of drug discovery and, in fact, can be layered into different stages during this course, including drug design, drug indication, drug repositioning, drug interactions, etc. For example, in early drug discovery, they can participate in the prediction of drug interactions or affinities with protein or miRNA targets for screening lead compounds, which can partly reduce the heavy workloads throughout HTS. Methodologically, these computational techniques can roughly be divided into two groups in light of whether the algorithms, themselves, demand a certain optimization process for generating intelligent models. For example, the Drutai tool demonstrated alongside this review was assembled as a result of a series of optimization iterations. Importantly, similarity-based inference techniques, many of which do not necessitate an optimization procedure for parameter estimation, are prevalently used in this field. As in Ref. [183], for example, the drug similarity is calculated as the weighted arithmetic mean of the clinical and structural similarities and the miRNA similarity is estimated based on the gene functional similarity and the cosine similarity. Yet another example is shown in Ref. [22] that the drug's Tanimoto similarity is employed in search of lead compounds. We found that the sequence-based prediction of DTIs has been in the groove by achieving 85–95% AUC values, implying that the current volume of DTIs in the DrugBank database suffices to generate deep learning models to be used in practice. In addition, it is very interesting to watch further cases or new modalities for validating the efficacy of the miRNA drugs discovered based on DeepsmirUD-predicted regulation types, disease-miRNA relationships, and connectivity scores.

Established studies have extensively benchmarked and discussed the biogenesis [245], structures [15], functions [42], targeting properties [4], silencing [246] of miRNAs, which, together, give insight into the pathogenesis of miRNA-regulated pathways [14]. These biological findings have yet to be tightly integrated with computational efforts. Starkly, the field of miRNAs is greatly devoid of functional annotations in their structures [247], due to a much more pronounced paucity of 3D native structures than those of proteins. The bulk of the ongoing efforts is being given to the design of covariation-based tools [248] for prediction [15]. From a limited number of case reports, we gained an understanding about the mechanism of the miRNA targeting that is specifically and selectively carried out in structured regions, like in the stem-loop hairpin secondary structure. Either or both the prediction-assisted and the experiment-led annotations of function sites are assumed to play a nonnegligible role in enabling the downstream analysis for computation-assisted structure-based drug design and discovery, provided that these computationally elaborated structures can be put on a par with those experimentally resolved ones. In this aspect, with many experimentally resolved miRNA structures becoming available, the information on specific functional sites (binding/interaction/inhibition) derived from these structures will be accumulated and can be used to train cutting-edge deep learning models, which will supply accurate prediction-assisted annotations of function sites in miRNAs on a large scale.

## Declaration of competing interest

The authors declare that they have no known competing financial interests or personal relationships that could have appeared to influence the work reported in this paper.

## Data Availability

Drutai is available at https://github.com/2003100127/drutai, which is used for predicting the interactions between drugs and protein targets
