# Supplementary material for: Small molecule-mediated targeting of microRNAs for drug discovery: Experiments, computational techniques, and disease implications
Source: Eur J Med Chem. 2023 Sep 5;257:115500. doi: 10.1016/j.ejmech.2023.115500 (PMC11554572; doi:10.1016/j.ejmech.2023.115500)
Supplement: Multimedia component 1 [file mmc1.docx]

***Supplementary Material***

**Small molecule-mediated targeting of microRNAs for drug discovery: experiments, computational techniques, and disease implications**

Jianfeng Sun^1,*,#^, Miaoer Xu^2,*^, Jinlong Ru^3^, Anna James-Bott^1^, Dapeng Xiong^4,5^, Xia Wang^6,#^, Adam P. Cribbs^1,#^

^1^ Botnar Research Centre, Nuffield Department of Orthopedics, Rheumatology and Musculoskeletal Sciences,

University of Oxford, Oxford OX3 7LD, UK

^2^ Department of Biology, Emory University, Atlanta, GA 30322, USA

^3^ Chair of Prevention of Microbial Diseases, School of Life Sciences Weihenstephan, Technical University of

Munich, Freising 85354, Germany

^4^ Department of Computational Biology, Cornell University, Ithaca, NY 14853, USA

^5^ Weill Institute for Cell and Molecular Biology, Cornell University, Ithaca, NY 14853, USA

^6^ College of Animal Science and Technology, Northwest A&F University, Yangling 712100, China

^*^ These authors wish to be known as equally contributed.

^#^ Correspondence: [adam.cribbs@ndorms.ox.ac.uk](mailto:adam.cribbs@ndorms.ox.ac.uk); [xiawang@nwafu.edu.cn](mailto:xiawang@nwafu.edu.cn); [jianfeng.sun@ndorms.ox.ac.uk](mailto:jianfeng.sun@ndorms.ox.ac.uk)

## Drutai: sequence-based prediction of DTIs

In order to understand to what extent DTIs can accurately be predicted based on the current DTI data from DrugBank, we used various deep learning algorithms to train predictive models and tested them in a few scenarios. The key procedures for generating the models and comparing the performance are presented below.

**Method**. We employed a total of 12 deep learning algorithms whose performance has been comprehensively examined in our previous work concerning SM-miR regulation type prediction. The settings of all of these algorithms are the same as used previously in [1]. Similarly, we also constructed an ensemble model, termed Drutai, by averaging out the predictions of individual models as the final decision about whether a drug-target pair is an interaction.

**Data processing**. In addition to removing DTIs that are repeated or have protein sequences represented with non-standard amino acid symbols, we also retained DTIs that involve proteins with a length of no more than 3000 (see **Table 4**). This is because these proteins are typically underrepresented in DTIs, which could potentially cause overfitting [2]. As summarized in **Supplementary Table 1**, known DTIs in both training and test datasets consist of two components, which are derived from the counterparts belonging to the FDA-approved and experimental drug categories, respectively. These DTIs were spilt and merged for training and test according to two ratios 9:1 (FDA-approved) and 2:1 (experimental), respectively. The training and test DTIs of FDA-approved drugs and experimental drugs are then put into four sets, which are named Train, Test, TrainExp, and TestExp, respectively. Similar to the testing of prediction models used in [3], we partitioned part of the test DTIs of experimental drugs into two sets that contain DTIs formed with novel drugs and targets in TestExp versus both Train and TrainExp, respectively. The novel drugs/targets have not been seen by the prediction models during the training stage. These two datasets can allow us to gain an understanding of the prediction performance when an input target/drug is considered as newly identified. These DTIs are treated as positive samples that are known to be in interaction. It is well-believed that this field in terms of DTI data resources greatly lack negative samples that are known to be in non-interaction. As mentioned earlier in this review, to overcome this problem, we use the guilt-by-association approach. In practice, obtaining negative samples using this approach can be a challenging task due to the significant computation power required to generate two large networks - the drug-drug similarity network using a Tanimoto coefficient cut-off 0.6, and the protein-protein similarity network using a sequence identity cut-off 0.4. According to our calculations shown in **Supplementary Table 2**, drug-drug and protein-protein pairs that appear in the two networks range from millions of pairs to tens of million pairs. The approved and experimental drugs for calculating the networks were extracted from the DrugBank-derived SDF file containing full drug structures. To balance between positive and negative samples in training and test datasets, we used equal number of negative samples from the guilt-by-association-derived drug-target pairs using the uniform distribution, respectively. Machine learning performance may be confounded by the datasets used. For this reason, we compared the performance of models trained on two version of the DrugBank databases. Finally, by combining these positive and negative samples, we obtained 29,198 (version: 5.1.9) and 28,674 (version: 5.1.8) pairs for training deep learning models.

**Feature preparation**. The compositional features of an input protein sequence include amino acid composition (1-mer), di-amino acid composition (2-mer), and tri-amino acid composition (3-mer), thus leading to a feature vector with a length of 8420. Then, we used PyBioMed [4] to generate a CTD (composition, transition, and distribution) feature vector with a length of 147. The CTD features were first proposed by Dubchak et al., describing the physicochemical properties of the protein, such as hydrophobicity and polarizability (for details, see [5,6]). From an input chemical compound structure, we extracted two kinds of fingerprints, namely, the Morgan fingerprints with a length of 1024 and the Torsion fingerprints with a length of 2048. In addition, we picked 197 chemical descriptors using RDKit [7], which include logP, molar refractivity, and the number of heavy atoms for example. The full list of these descriptors can be found at <https://github.com/2003100127/drutai>. These protein and compound features are finally concatenated into a single one vector with a length of 11,836.

**Performance validation.** To assess the generalization ability of the trained models, we included two additional independent datasets, in addition to the aforementioned test datasets. These datasets comprised DTIs, each with a novel target/drug that was never used and seen by deep learning models during the training and optimization processes. This allowed for an objective evaluation of model performance. Our main evaluation metrics were the AUC and the AUCPR, as they examined performance thoroughly [8]. For instance, the AUCPR value is generated by comparing precision against recall at all thresholds. The AUC measures the area under the receiver operating characteristic (ROC) curve [9], while the AUCPR measures the area under the Precision-Recall curve [10].

Overall, the deep learning models, together with the final ensemble model, show a competitive performance in the DTI prediction (**Supplementary Figures 1** and **2**). We used over 400 training epochs to visualise the AUC values (**Supplementary Figure 1a and 1b**), which demonstrates that deep learning algorithms can capture the characterization of drug-target interactions and non-interactions using only protein sequence-based information. This is evident from the minor variations in AUC values for almost all the algorithms. Notably, the majority of the algorithms exhibited long-term stability in prediction performance based on the test DTIs of the FDA-approved category. Moreover, several algorithms showed a steady increment in AUC values along epochs based on the test DTIs of the experimental category. These observations are confirmed by R-square values of above 0.8 and statistically significant p-values, implying that overfitting does not occur seemingly. As is presented in Figure 5, the ROC and Precision-Recall curves show that there are no significant differences between the model performance using the two versions of the DrugBank databases. On the test DTIs of FDA-approved drugs, the performance of individual models is examined to vary in a range from 0.840 to 0.930 for both AUC and AUCPR values. By contrast, individual models show a quite divided performance on the test DTIs of experimental drugs, such that all models involving residual units (MobileNetV2, ResNet18, ResNet50, SCAResNet18) plus LSTMCNN yield AUC values ranging from 0.810 to 0.850 (similar to AUCPR values, **Supplementary Figure 2a and 2b**) but the rest of models have AUC values ranging from 0.700-0.800. Using the two datasets of novel targets and novel drugs, the performance can rise up to AUC values of 0.744 and 0.724 at maximum when these models are introduced to discover potential new therapeutic targets and new drugs (**Supplementary Figure 5c and 5d, Figures 5 and 6**). It is apparent that Drutai and the MobileNetV2 model can yield more convincing results.

**Supplementary Table 1**. Number of known DTIs in DrugBank for training, test, and validation.

| **Version** | **Approved** | | **Experimental** | | | | **Total train** |
| --- | --- | --- | --- | --- | --- | --- | --- |
|  | **Train** | **Test** | **Train** | **Test** | **Novel target** | **Novel drug** |  |
| 5.1.8 | 8799 | 977 | 5538 | 2768 | 794 | 1514 | 28,674 |
| 5.1.9 | 9079 | 1008 | 5520 | 2760 | 962 | 1503 | 29,198 |

**Supplementary Table 2**. Number of drug-target pairs generated by the guilt-by-association approach.

| **Version** | **Type** | **Similarity pair** | **Using drug-drug similarity network** | | **Using target-target similarity network** | |
| --- | --- | --- | --- | --- | --- | --- |
|  |  |  | **below<0.6** | **above>0.6** | **below<0.4** | **above>0.4** |
| 5.1.8 | Approved | 3,557,778 | 4,500,558 | 1024 | 3,734,433 | 920 |
|  | Experimental | 19,080,753 | 11,963,292 | 5965 | 9,732,605 | 4546 |
| 5.1.9 | Approved | 3,714,175 | 4,707,550 | 1261 | 4,401,686 | 957 |
|  | Experimental | 19,303,791 | 11,996,082 | 5910 | 9,663,346 | 3877 |


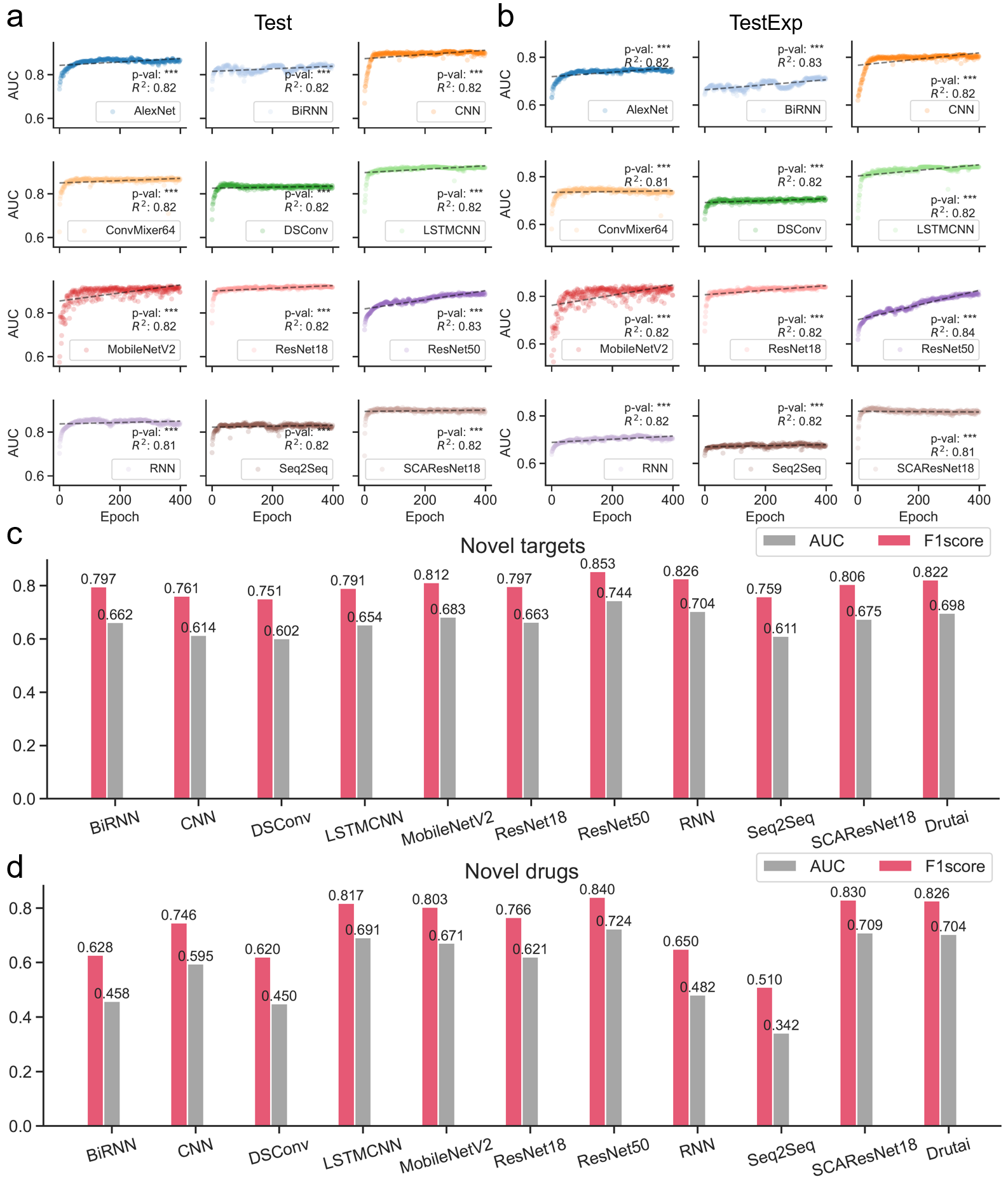


**Supplementary Figure 1**. Performance comparison. AUC values of various deep learning models changing with 400 epochs on the (a) Test and (b) TestExp datasets. (c) and (d) show the AUC and F1score performance on DTIs formed with novel targets or novel drugs, respectively.


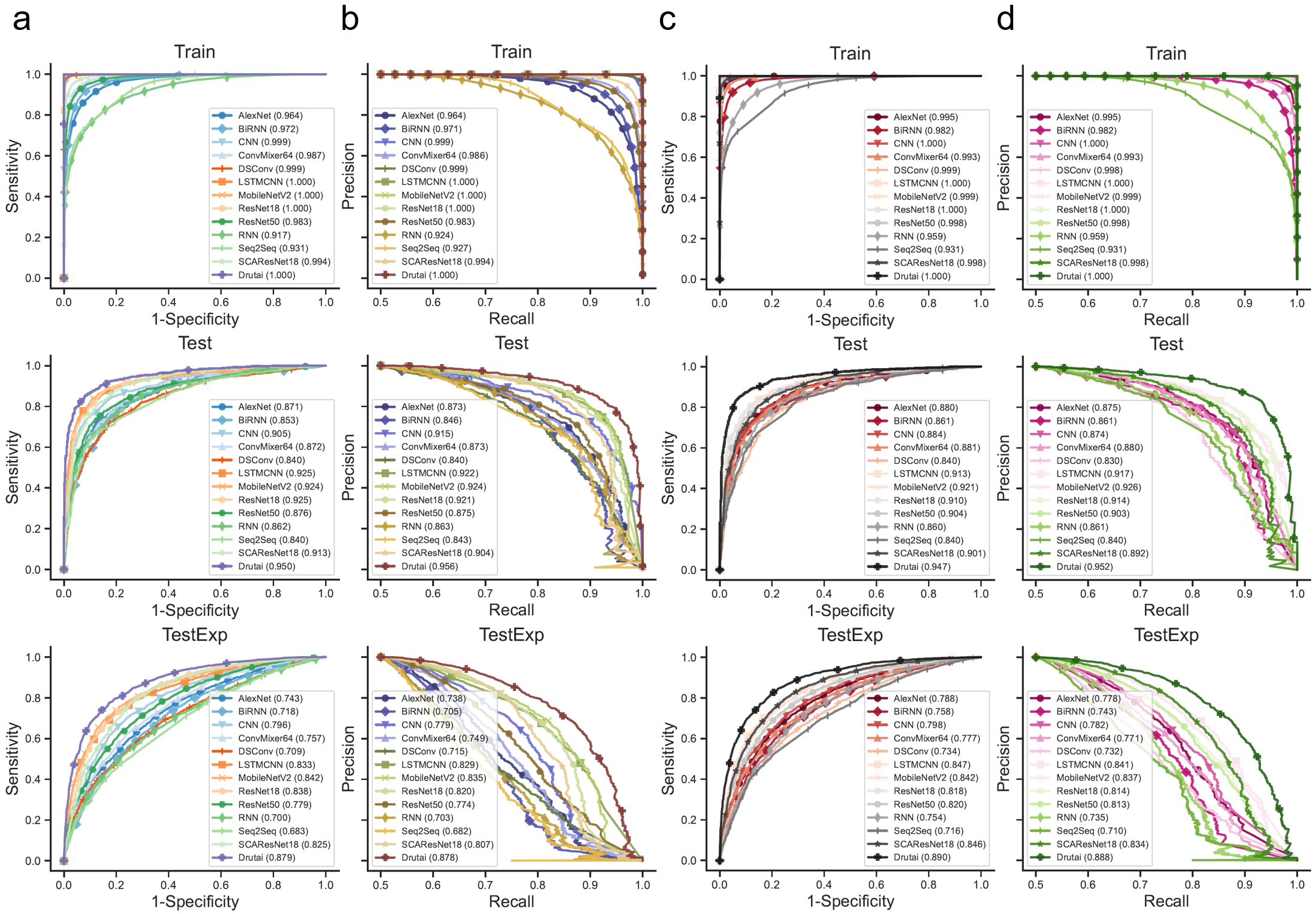


**Supplementary Figure 2**. Performance comparison using threshold-free evaluation metrics. (a) ROC and (b) Precision-Recall curves on the Train, Test, TestExp datasets generated based on the version 5.1.8 of the DrugBank database. (c) ROC and (d) Precision-Recall curves on the Train, Test, TestExp datasets generated based on the version 5.1.8 of the DrugBank database.

[1] J. Sun, J. Ru, L. Ramos-Mucci, F. Qi, Z. Chen, S. Chen, A.P. Cribbs, L. Deng, X. Wang, DeepsmirUD: Prediction of Regulatory Effects on microRNA Expression Mediated by Small Molecules Using Deep Learning, Int J Mol Sci. 24 (2023). https://doi.org/10.3390/ijms24031878.

[2] L. Prechelt, Automatic early stopping using cross validation: quantifying the criteria, Neural Networks. 11 (1998) 761–767. https://doi.org/https://doi.org/10.1016/S0893-6080(98)00010-0.

[3] M. Wen, Z. Zhang, S. Niu, H. Sha, R. Yang, Y. Yun, H. Lu, Deep-Learning-Based Drug–Target Interaction Prediction, J Proteome Res. 16 (2017) 1401–1409. https://doi.org/10.1021/acs.jproteome.6b00618.

[4] J. Dong, Z.-J. Yao, L. Zhang, F. Luo, Q. Lin, A.-P. Lu, A.F. Chen, D.-S. Cao, PyBioMed: a python library for various molecular representations of chemicals, proteins and DNAs and their interactions, J Cheminform. 10 (2018) 16. https://doi.org/10.1186/s13321-018-0270-2.

[5] I. Dubchak, I. Muchnik, S.R. Holbrook, S.H. Kim, Prediction of protein folding class using global description of amino acid sequence., Proceedings of the National Academy of Sciences. 92 (1995) 8700–8704. https://doi.org/10.1073/pnas.92.19.8700.

[6] I. Dubchak, I. Muchnik, C. Mayor, I. Dralyuk, S.-H. Kim, Recognition of a protein fold in the context of the SCOP classification, Proteins: Structure, Function, and Bioinformatics. 35 (1999) 401–407. https://doi.org/https://doi.org/10.1002/(SICI)1097-0134(19990601)35:4<401::AID-PROT3>3.0.CO;2-K.

[7] A.P. Bento, A. Hersey, E. Félix, G. Landrum, A. Gaulton, F. Atkinson, L.J. Bellis, M. de Veij, A.R. Leach, An open source chemical structure curation pipeline using RDKit, J Cheminform. 12 (2020) 51. https://doi.org/10.1186/s13321-020-00456-1.

[8] Y. Yuan, W. Su, M. Zhu, Threshold-Free Measures for Assessing the Performance of Medical Screening Tests, Front Public Health. 3 (2015) 57. https://doi.org/10.3389/fpubh.2015.00057.

[9] K. Boyd, K.H. Eng, C.D. Page, Area under the Precision-Recall Curve: Point Estimates and Confidence Intervals BT  - Machine Learning and Knowledge Discovery in Databases, in: H. Blockeel, K. Kersting, S. Nijssen, F. Železný (Eds.), Springer Berlin Heidelberg, Berlin, Heidelberg, 2013: pp. 451–466.

[10] T. Saito, M. Rehmsmeier, The Precision-Recall Plot Is More Informative than the ROC Plot When Evaluating Binary Classifiers on Imbalanced Datasets, PLoS One. 10 (2015) e0118432. https://doi.org/10.1371/journal.pone.0118432.
